# Functionalized graphene oxide-based electrode material for the potentiometric detection of codeine phosphate in commercial cough syrups for forensic applications

**DOI:** 10.1038/s41598-026-44986-4

**Published:** 2026-04-08

**Authors:** Ephrin S, Jebasingh Bhagavathsingh, Sneha Abraham, M Nesasudha, Doondi Kumar Janapala

**Affiliations:** 1 Department of Applied Chemistry, Karunya Institute of Technology and Sciences, Coimbatore, 641114 Tamilnadu India; 2 Department of Chemistry, School of Energy Technology, Pandit Deendayal Energy University, Raysan, 382426 Gandhinagar, Gujarat India; 3 Centre for Nanosciences and Genomics, Division of Physical Sciences, Karunya Institute of Technology and Sciences, Coimbatore, 641114 Tamilnadu India; 4 Department of Electronics and Communication Engineering, Karunya Institute of Technology and Sciences, Coimbatore, 641114 Tamilnadu India; 5 Department of Electronics and Communication Engineering, Vishnu Institute of Technology, Bhimavaram, 534202 Andra Pradesh India

**Keywords:** Illicit drug, Codeine Phosphate, Bromophenol Blue, UV Spectrometry, Voltammetry, Chemistry, Materials science, Nanoscience and technology

## Abstract

The current trend in the detection of forensic or banned drugs has attracted many researchers to the development of engineered nanoparticle-based sensor devices for the rapid detection of these drugs at affordable costs. Herein, we report the development of functionalized graphene oxide active nanosheets (GO-Bpb) for the detection of codeine phosphate in commercially available cough syrup using potentiometric and colorimetric methods. The active material was characterized for the successful intercalation by spectroscopic and microscopic techniques. The sensor strip was fabricated by spin-coating the active material onto Whatman filter paper, incorporating copper electrodes on both sides, with a Teflon separator. Upon dipping into the various concentrations of the test solutions, the variation in the voltage was measured due to the analyte interactions on the active material until the lowest possible concentration. For the colorimetric method, the stock solution of GO-Bpb vs. various concentrations of codeine was measured by UV-Vis spectroscopy for the quantification of the analyte at a lower concentration. It resulted in a limit of detection (LoD) of 0.0412 mg/mL. The cost-effective, reproducible strip-based detection paves the way to achieve a more sensitive and selective method with a LoD of 0.0018 mg/mL for the on-site detection of illicit drugs during crime scene investigation.

**Supplementary Information:**

The online version contains supplementary material available at 10.1038/s41598-026-44986-4.

## Introduction

Illicit drugs are substances that are illegal to use, produce, or distribute, often due to their potential for abuse and harm. The detection of abused drugs at crime scenes is a critical aspect of forensic science for criminal investigations and legal proceedings^1^. With an increasing conduct of drug-related offenses^2^. Innovative development of a rapid device for the on-site detection of used or involved illicit drugs has become essential for forensic investigation^3^. The different illegal drugs are categorized based on their origin, chemical structure, and effects on the human body. The primary categories include cannabis, opiates, cocaine, amphetamine-type substances, and new psychoactive substances (NPS)^4^. Pharmaceutical drug abuse encompasses a variety of substances, such as Benzodiazepines and over-the-counter (OTC) medications, that are misused for their sedative effects^5^. Illicit drugs like codeine and dextromethorphan are the prime ingredients in the commercially available cough syrup or tablets^6^. Alcohol and Nicotine are frequently combined with the prescribed drugs, increasing the risk of severe health complications^7^. Prescription drug abuse is a growing concern globally^8,9^. Long-term abuse results in significant cognitive impairments, fatigue, and behavioural changes, including aggression and impulsivity^10^. Although various advanced analytical methods are highly sensitive for the detection, limiting the use for rapid field screening due to sophisticated instrumentation and lab-based analysis^11^. Additionally, the development of portable drug detection devices has enhanced the ability of law enforcement to conduct on-site preliminary analyses, expediting the investigative process^12,13^. Codeine phosphate is one of the principal ingredients in cough syrups, with the combined effect of the associated molecules, and consumption with various drugs^14^. The prolonged use of codeine has been shown to adversely affect female reproductive health, leading to ovarian toxicity and dysfunction^15^.

**Fig. 1:**
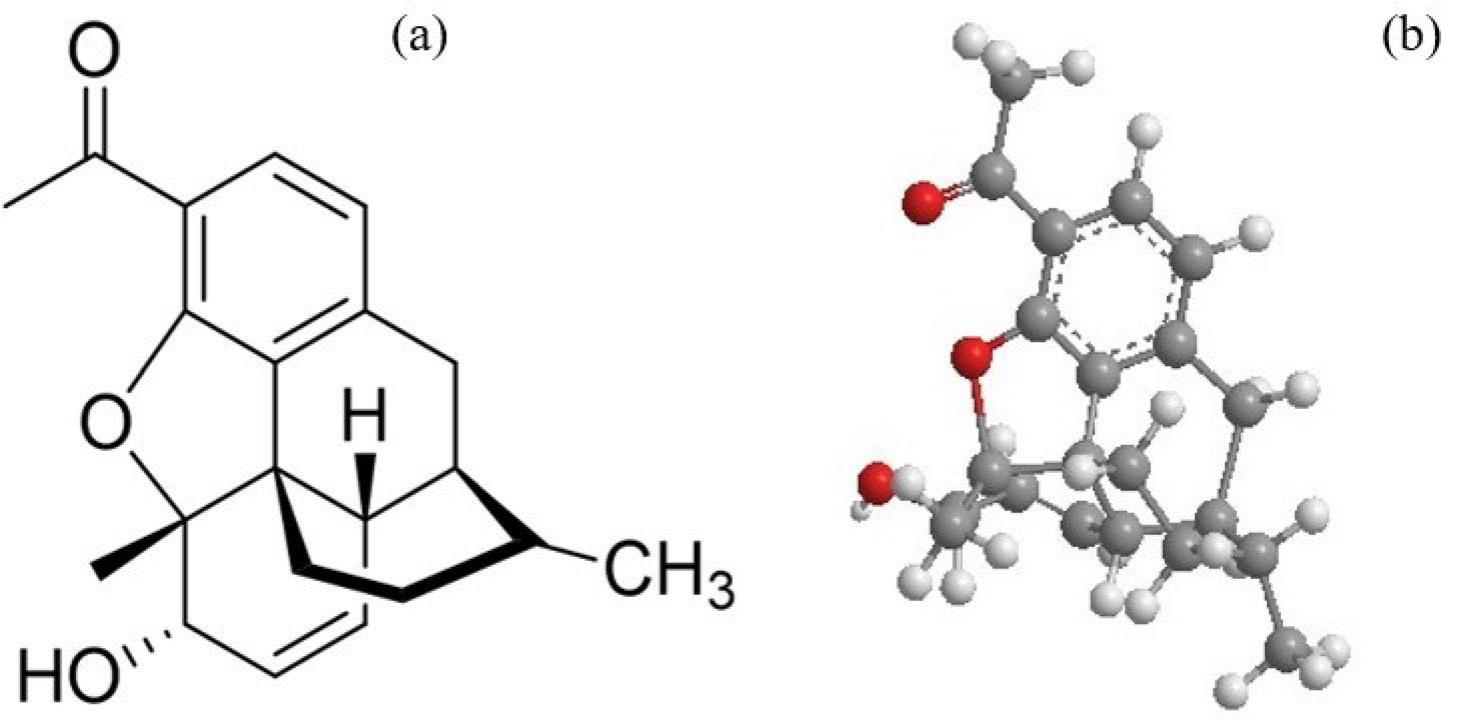
**a** Chemical structure of Codeine, **b** 3D Molecular structure.

In Fig. 1a, the chemical structure of codeine and Fig. 1b, the 3D molecular structure, as it is illegal to use another derivation, Codeine phosphate is an opioid used in some cough syrups to suppress cough and mild pain. It carries a high potential for abuse when taken in higher doses^16^. The detection of codeine phosphate can be achieved through various innovative methods, such as Electrochemical Sensing using Graphene Quantum Dots (GQDs) Modified screen-printed carbon electrodes, utilizing GQDs for codeine detection in biological fluids and soft drinks, with LoD 0.21 µM^17^. Essam et al. reported the development of potentiometric sensors fabricated using gold nanoparticles, which showed high sensitivity, slope, and response time^18^. Karim et al. developed an accurate method for the simultaneous detection of codeine with other drugs using the RP-HPLC technique. But when it lacks the isolation of the sample, on-site detection^19^. These studies of strip-based sensors facilitate the detection of codeine phosphate from the other pharmaceutical ingredients. The paper-based sensor device was reported with the claim of a simple, increased shelf life, and testing duration with improved reproducibility^20^. Shende et al. have developed a strip using SER flow separation, which has insufficient sensitivity to detect codeine and fentanyl in biofluids. To amplify the Raman signals, strips are incorporated with gold or silver nanoparticles as they increase the surface area to improve the sensitivity^21^. Abd-Rabboh et al. have reported a cost-effective paper-based device for the potentiometric detection of Pholcodine on chemically reduced Graphene oxide, as it increases the sensitivity and reveals a detection limit of 0.04 *µ*g mL^− 1^^22^. An electrochemical sensor using Carbon paste electrodes (CPE) modified with multi-walled carbon nanotubes (MWCNT) and NiO nanoparticles for the determination of codeine in real samples, with limits of detection (LOD) of 0.015 µM^23^. An aptamer-based graphene sensor has been developed for the detection of codeine, achieving a detection limit of 3.2 pM and a linear range of up to 900 nM^24^. Nanocomposites of graphene with metal oxides, such as CoFe₂O₄, have been used to fabricate modified carbon paste electrodes for the detection of codeine, with a detection limit of 0.011 µM and a linear range of 0.03–12.0 µM^25^. A functionalized γ-alumina/graphene oxide (γ-NGO) composite was effective for copper ion detection, exhibiting a low detection limit (4.0 × 10^− 8^ M) and fast response time (15s)^26^.

Currently, the 2D material composites are used to develop potentiometric sensors to improve the sensitivity, selectivity, and identification of illicit drugs in complex crime scene environments^27–31^. However, the onsite detection remains challenging due to cost, sample preparation, and complex instrumentation. Paper based sensor are reported with simple operation and portability.

Bromophenol blue is an anionic dye with a sulfonate group, which can form ion-pair complexes with the protonated amine in codeine phosphate. These charge-based drug detections are widely used by spectrophotometric methods for sensitive identification. For example, Bromophenol Blue (Bpb) was used for the determination of pharmaceutical drug cefixime trihydrate through a charge transfer interaction for high sensitivity and low detection limit^32^. When it is intercalated with graphene oxide, the analyte absorption enhances due to the high surface area and π-π conjugated structure.

Herein, we report a rapid, cost-effective method developed using bromophenol blue functionalized graphene oxide nanosheets-based active material for the detection of codeine phosphate in commercially available cough syrups. The sensor strip was fabricated using the spin coating of graphene-based bromophenol active material on the Whatman filter paper with copper electrodes on both sides, with a Teflon separator. The concept of screen-printed strips with the active materials for the detection of various analytes paves the way to the real-time detection of analytes for the first-time information about the presence of analytes in crime scenes.

## Materials and methods

### Materials

Triprolidine Hydrochloride and Codeine Phosphate containing Cough syrup (Tossex) was purchased commercially. Each 5 mL of Tosses contains Triprolidine Hydrochloride (1.25 mg) and Codeine phosphate (10 mg), with the concentration of codeine phosphate being 2 mg/mL. The control experiment with the codeine molecule was performed to assess the interference of Triprolidine Hydrochloride in the process of detection. No significant colour change was observed with the additive molecules. Bromophenol Blue [pH of 3.0-4.6] and Lucas Reagent were purchased from M/s. Merck India. Anhydrous Zinc Chloride (ZnCl_2_) in conc. HCl was obtained from Himedia chemicals. The triple-distilled water was used for the dilution of forensic drugs and other chemicals involved in the study. The chemicals KMnO_4_, NaNO_3_, Conc. H_2_SO_4_, H_2_O_2_ (30%), Ethanol, Acetone, and Diethyl ether were purchased from LOBA chemicals.

### Instruments

Ultrasonicator from LABMAN Scientific Instrument. Model No- LMUC-9 of 8 L capacity with the frequency of 40 kHz was used. The ultrasonic power of 200 W was applied to the tank size of 300 × 240 × 150 mm. The operational temperature of 30–65 °C was used in the ultrasonicator. Remi R-8 C Laboratory Centrifuge Maximum Speed: 6000 RPM, Maximum RCF (Relative Centrifugal Force): ~5070 × g, Capacity: Up to 400 ml, Rotor Options: Swing-out and angle rotors available, Timer: 0–99 min with digital display, Control System: Microprocessor-based control, Safety Features: Lid interlock, imbalance detection, and emergency lid opening, which was used to isolate the functionalized graphene oxide nanosheets.

High-precision multimeter, Agilent Technologies, USA, used to test the output of the electronic testing and laboratory applications. Agilent (Keysight) digital multi-meters (DMMs), Agilent 34,401 A Digital Multi meter (6.5 Digits), Display: 6.5-digit resolution, DC Voltage Range: 100 mV to 1000 V, AC Voltage Range: 100 mV to 750 V, DC Current Range: 10 µA to 3 A, AC Current Range: 10 µA to 3 A, Resistance: 100 mΩ to 100 MΩ, Frequency Measurement: 3 Hz to 300 kHz, Basic DC Accuracy: 0.0035%, Interface: GPIB, RS-232 and Application: High-precision laboratory and industrial testing to measure in voltage.

For the lower-level detection, the quantitative analysis of codeine phosphate is determined by UV spectroscopy. These methods leverage UV-visible spectrophotometry to accurately measure codeine phosphate concentrations in different concentrations. JASCO V-770 UV-Vis-NIR Spectrophotometer Specifications: Double-beam with Czerny-Turner mount monochromator. Grating: 1200 lines/mm (UV-Vis), 600 lines/mm (NIR), and Detector: PMT (Photomultiplier Tube) for UV-Vis; PbS (Lead Sulfide) for NIR, with a range 190–2700 nm (UV-Vis-NIR) was used.

The XRD patterns for polymeric materials were recorded on a Shimadzu XRD- 6000 Powder X-Ray diffractometer at 40 kV voltages and 30 mA current. All the spectra were acquired at a pressure using an ultra-high vacuum with Al Kα excitation at 250 W. Scanning electron microscope (SEM) images were measured by the Hitachi S4800 field emission SEM system.

## Experimental

### Synthesis of Graphene Oxide (GO)

Graphene Oxide was prepared using the Hummers method as reported^33,34^. Briefly, 5 g of Graphite and 2.5 g of NaNO3 were mixed with 108 mL of H_2_SO_4_ and 12 mL of H_3_PO_4,_ and stirred in an ice bath for 1 h. Next, 15 g of KMnO_4_ were slowly added so that the temperature of the mixture remained below 5 °C. The suspension was then reacted for 2 h in an ice bath and stirred for 60 min before again being stirred in a 40 °C water bath for 24 h. The temperature of the mixture was adjusted to 10 °C for 60 min while water was added continuously. Deionized water was further added so that the volume of the suspension was 500 mL, and 15 mL of H_2_O_2_ was added for 1 h. The reaction product was centrifuged and washed with deionized water (two cycles), ethanol (two cycles), Acetone (two cycles), and Diethyl ether (two cycles). In each step, the material was redispersed and centrifuged to remove impurities. and a free-flow powder was formed.

### Synthesis of bromophenol blue functionalized Graphene Oxide Nano sheets (GO-Bpb)

Bromophenol blue was intercalated on the basal planes of GO Nano sheets using high dilution techniques. The functionalized GO was prepared by taking 1 g of well-dispersed GO nanosheets in 500 mL of water under sonication for 30 min at room temperature. To the mixture, 1 gm of bromophenol blue in 300 mL of tetrahydrofuran was added slowly at room temperature with vigorous stirring. The resulting solution was stirred for 48 h to complete the intercalation. The aliquot was tested for the presence of bromophenol blue by Thin-Layer Chromatography, and the absence of the intercalant confirms the efficient functionalization on the GO plane. The solution was centrifuged to isolate the black residue and washed with ethanol and acetone, followed by diethyl ether. The functionalized GO nanosheets were obtained as a black, free-flowing solid. A highly diluted dispersed GO was used for the preparation of GO-Bpb to produce effective exfoliation and interaction with bromophenol blue. Whereas, preliminary trials showed that lower dilutions resulted in aggregation of GO sheets.

### Device fabrication

#### Strip preparation using bromophenol blue and codeine phosphate

The cost-effective strip model was designed for the detection of codeine phosphate in commercial cough syrups. When the active material was in contact with the different concentrations of codeine, the corresponding responses in mV were observed. Initially, the solution of bromophenol blue (0.025 mg/mL) in deionized water was spin-coated on the surface of a 15 mm x 40 mm size Whatman 2Å filter paper and dried in a desiccator for 30 min. The copper electrode size of 4 mm x 50 mm was placed on the surface of the coated paper. The non-conducting polyester film as a separator was placed between the two half electrodes with a size of 15 mm x 40 mm, with the copper strip covered with the same size of the graphite spin-coated paper. These electrodes were wrapped with a nozzle at the bottom for the entry of the test solutions. The copper electrodes were connected to the testing equipment for the detection of codeine phosphate present in the test solution for the measurement of the variations with various concentrations of the test solutions. The same methodology was followed with bromophenol blue solutions of 0.025 mg/mL, 0.05 mg/mL, 0.1 mg/mL, 0.15 mg/mL, and 0.2 mg/mL, respectively.

### Strip preparation using bromophenol blue intercalated Graphene Oxide material (GO-Bpb)

For the preparation of the strip, the concentration of GO-Bpb was selected based on preliminary optimized studies, which were 1 mg/mL. The concentration higher than the selected concentration produce thick film and an unstable response. The concentration lower than the selected concentration produce very low voltage response.

Briefly, the bromophenol blue intercalated GO nanosheets (1 mg/mL) were well dispersed in deionized water using an ultrasonicator. The as-prepared solution was spin-coated on the surface of a 15 mm x 40 mm size Whatman 2Å filter paper and dried in a desiccator for 30 min, and the same was performed with the graphite. On top of the coating, a copper electrode with a size of 4 mm x 50 mm was placed. The non-conducting polyester film as a separator was placed between the two half electrodes, with a size of 15 mm x 40 mm, with the copper strip covered with the same size of the graphite spin-coated paper. These electrodes were wrapped with a nozzle at the bottom for the entry of the test solutions. The two copper electrodes were connected to the testing equipment for the detection of codeine phosphate in the test solution for the measurement of the variations with various concentrations of the test solutions.

### Test solution for colorimetric studies

For the colorimetric detection of codeine, commercially available cough syrup was utilized to identify codeine phosphate as a forensic drug. The codeine phosphate (Tossex brand, 2 mg/mL) was diluted by the serial dilution method to prepare various concentrations of 0.1 mg/mL, 0.2 mg/mL, and 0.4 mg/mL in deionized water. To these solutions, 0.25 mg of bromophenol blue in 10 mL of deionised water was added to develop the coloured solution according to the concentration. As the concentration of codeine phosphate increased, the colour of the solution changed from pale purple to dark purple. These colorimetric solutions were subsequently analysed using UV-Vis spectroscopy.

On the other hand, the low concentrations of bromophenol blue, ranging from 0.00625 mg/mL, 0.0125 mg/mL, 0.025 mg/mL, 0.05 mg/mL, and 0.1 mg/mL of deionised water, were prepared using the serial dilution technique. To these blue coloured solutions, codeine phosphate in 0.2 mg/mL of deionised water was added at room temperature to yield the pale purple to dark purple colour. These coloured solutions were used for the UV-Vis spectral analysis.

### Result and discussion

**Scheme 1:**
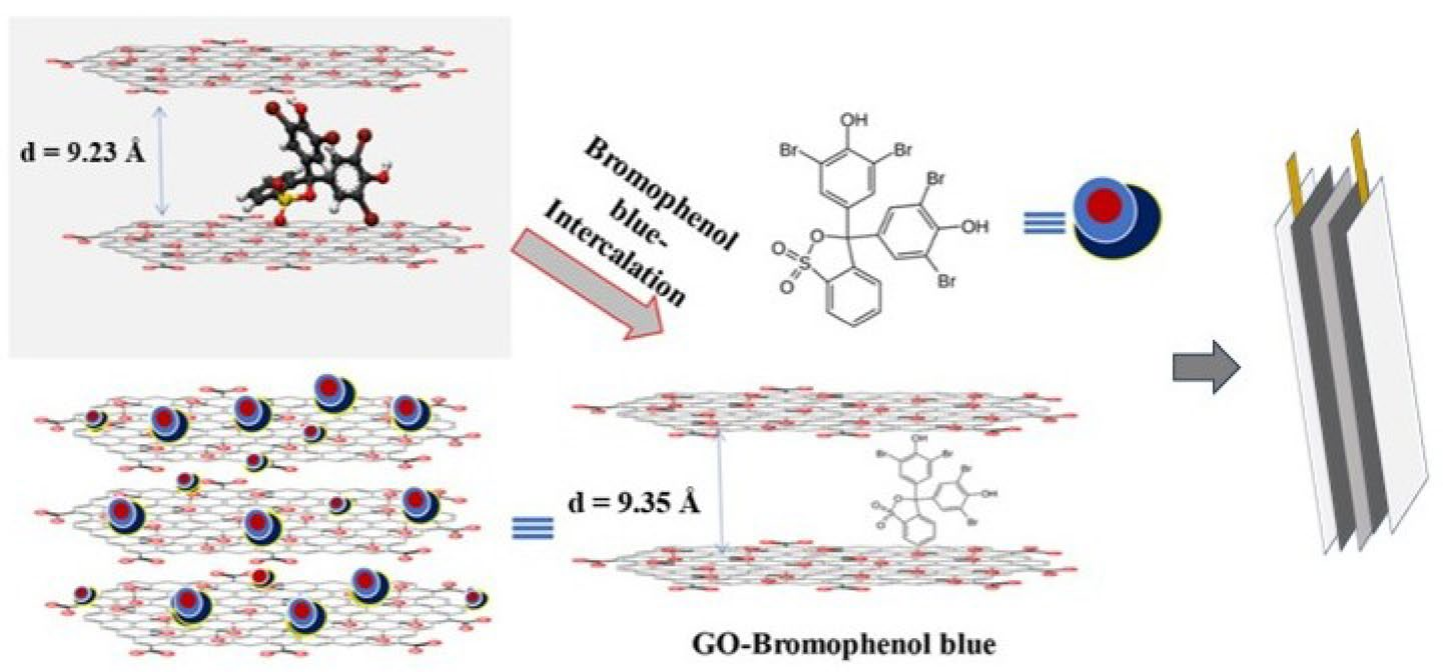
The schematic representation of the development of a strip based on Bpb intercalated GO nanosheets as an active material for the detection of codeine.

The codeine phosphate-containing cough syrup was investigated as a standard to explore the detection of the molecule present in the commercial entities using a convenient prototype prepared by the functionalized graphene oxide (*f*GO) nanosheets. Bromophenol blue was used as an indicator molecule to detect the presence of codeine. Additionally, the bromophenol blue was intercalated on the surface of the graphene oxide nanosheets by the high dilution technique using dual solvent systems. The un-intercalated material was washed with the various polarities of the solvent to remove the intercalated nanosheets and obtained as a free-flow black solid. The intercalated GO nanosheets were confirmed by the spectroscopic and microscopic techniques. The as-prepared material was used as an active electrode material for the detection of codeine, along with the graphite material and two copper leads for the measurement. The highly sensitive potentiostat was used to measure the difference in millivolts when the test solution was in contact. The device fabrication with the functionalized graphene oxide and its performance are shown in Scheme 1.

### XRD pattern analysis

XRD was used to analyse the intercalation of bromophenol blue on the surface of the GO basal planes and its diffraction patterns, which impact the interlayer *d*-spacings and the interaction of the analyte with the intercalant for further morphological changes in its nanostructure of the prepared material (Fig. 1a). The diffraction peak at 2θ value of 9.44 deg with the interlayer *d*-spacing of 9.35 Å corresponds to the functionalized of bromophenol blue through van der Waals interactions on the surface of the GO basal planes. In comparison with graphene oxide nanosheets, the diffraction peak shows at 9.56 ° with the interlayer *d*-spacing 9.23 Å, confirming the successful intercalation of bromophenol blue in between the GO-nanosheets by enhancing the interlayer *d*-spacing.

### FT-IR analysis

**Fig. 2:**
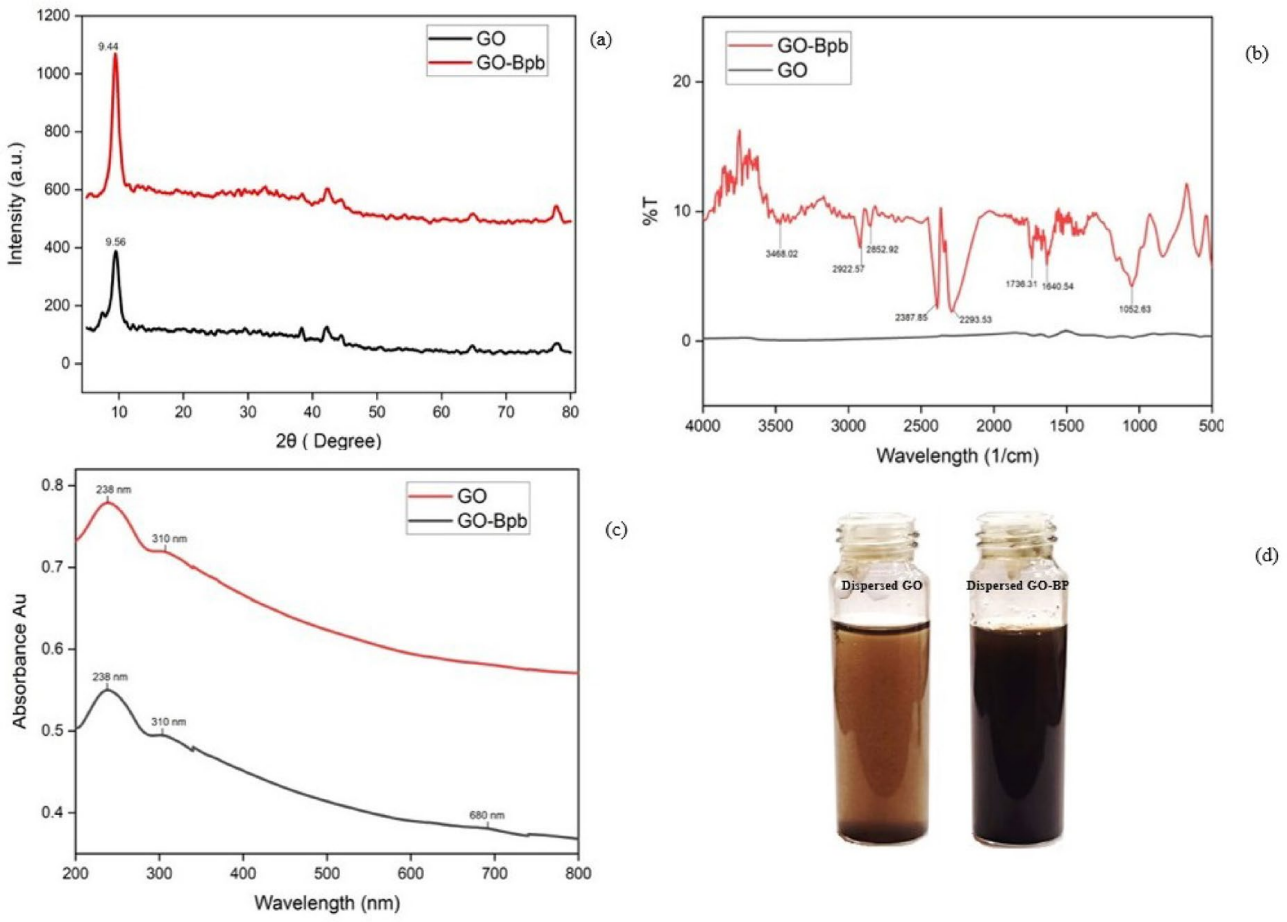
**a** XRD Pattern of GO and GO-Bpb, **b** FT-IR spectrum of GO and GO-Bpb, **c** UV-VIS spectrum of GO, GO-Bpb, and **d** Pictorial representation of the dispersed solution of GO and GO-Bpb.

The functional groups present in the GO Nano sheets upon functionalization were analysed by FT-IR spectrum (Fig. 2b). The broad peak at 3394 cm^− 1^ corresponds to the stretching vibrations of -OH moieties, and the characteristic peak at 2921.12 cm^− 1^ was attributed to the C-H stretching vibration existing in the aromatic rings. The peak at 1739 cm^− 1^ was due to the C = O stretching vibrations from the terminal acid moieties. The C = C bond stretching vibration shows at 1626 cm^− 1,^ and the peak at 1050 cm^− 1^ corresponds to the C-O bond vibrations.

### UV-VIS sSpectroscopy analysis

The UV spectrum of GO shows a characteristic sharp absorption peak at ~ 238 nm, which is attributed to the π → π* transition of the sp^2^ C = C bond (Fig. 2c), while the broad peak shoulder at ~ 310 nm is assigned to the n → π* transition of the C = O group. These features confirm the presence of oxygen-containing functional groups. After the intercalation of bromophenol blue to GO, a band appears at 680 nm corresponding to absorption by the bromophenol blue. This band is compared with the retention of the GO absorption feature, indicating the successful intercalation of Bpb in the GO nanosheet and increasing the sensing performance.

### SEM analysis

The surface morphology of pristine GO and the functionalised GO with bromophenol blue was analysed by SEM analysis. As in the figure S1a, the pristine GO with layered and wrinkled sheets indicates the formation of exfoliated graphene oxide nanosheets. After intercalation of BpB onto the GO nanosheets, the material exhibits a more aggregated and folded structure. In the figure S1b, an irregular and clustered domain observed at high magnification confirms the interaction of bromophenol blue molecules with the GO basal planes. The figure S1c at intermediate scale shows stacked and wrinkled confirms the formation of an aggregated structure after the Bpb incorporation. At the lower magnification in Figure S1d, the GO-Bpb is larger, and a stacked sheet-like cluster forming a continuous network improves the ion pair interaction and analyte absorption. These morphology changes confirm the successful functionalisation of GO with Bpb. The elemental composition of GO-Bpb (Figure S2) using the SEM-EDX confirms the presence of carbon (C) and Oxygen (O) from the graphene oxide, along with sulphur (S) and Bromine (Br) of the bromophenol blue group, confirming the successful functionalization of GO with Bpb. The detailed elemental composition obtained from the EDX analysis, including the weight percentages of the detected elements, is provided in Table S7 in the Supplementary Information.

### Strip fabrication for the detection of codeine

The two types of strips were fabricated using the active bromophenol blue molecule, as it has possible interactions with the codeine through ion-pair interactions, which facilitates the concentration-dependent colour change. Herein, we explored the strip prototype with various concentrations of Bpb against codeine and, additionally, various concentrations of codeine against the Bpb molecule. The variation in colours due to the interaction of codeine was analysed by UV-Visible spectroscopy and potentiometrically.

**Fig. 3:**
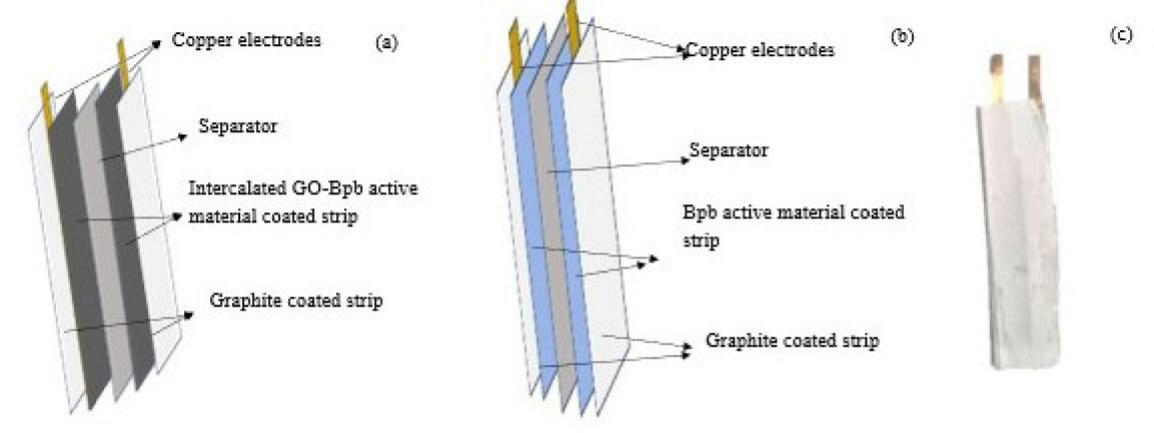
**a** Bpb intercalated GO nanosheet-based strip, **b** Bromophenol blue strip for the detection of Codeine Phosphate, **c** Pictorial representation of as-prepared bromophenol blue strip.

Firstly, the spin-coated strip was prepared using the various concentrations of bromophenol blue (0.025 mg/m, 0.05 mg/m, 0.1 mg/m, 0.15 mg/m, 0.2 mg/mL) combined with the graphite electrode material along with copper conducting strip separated by the polyester film for the detection of codeine phosphate (Fig. 3). The as-fabricated strip was tested with the various concentration of codeine phosphate and the potentiometric responses were recorded (Table S2). Attempts have been made to enhance the low-level detection of codeine using the Bpb molecule, and the efforts with the intercalated GO nanosheets were successful due to their large surface area. The second strip was fabricated using the Bpb intercalated GO nanosheets (Fig. 3a). Typically, 10 mg of Bpb intercalated GO nanosheets were dispersed in 10 mL of deionized water and spin-coated uniformly onto the Whatman filter paper. Each sample coated filter paper was kept dry for 30 min, and then the coated paper was cut into a size of 1.5 cm x 4 cm, and the copper strip with a size of 0.2 cm x 5 cm was placed on top of the active material. The protocols were repeated with the graphite materials, and these two coated papers were separated by the polyester separator. The as-prepared active material and the graphite material with a copper strip were sealed closely, as it was shown in Fig. 2b. The strip was dipped in the analyte, which was a concentration of 0.2 mg/mL of codeine phosphate (Fig. 3c). Then the two terminals were connected to the multimeter, and the readings were taken before and after dipping in the codeine solutions.

Selectivity mechanisms, the intercalated Bpb on the basal plane of GO nanosheets, may facilitate the formation of an ion-pair complex interaction with the intercalated Bpb, which may lead to the formation of π-π stacking arrangements with the GO nanosheets. Notably, the amount of Triprolidine Hydrochloride is much lower than the analyte content in the commercially available cough syrup, which will not interfere with the colour change. The added colorant was also quenched by the GO nanosheets. Therefore, the active material codeine present in the commercially available cough syrup forms a Hydrogen interaction with the phenolic -OH present in the Bpb.

### Potentiometric analysis

The potentiometric measurement was performed using agilent 34,407 A digital multimeter. The strip with a copper electrode was dipped in the test solution. Voltage was recorded after a 10–15 s of stabilization period. All the measurement was done under room temperature using deionised water. The variation of voltages due to the ion pair interaction of Bpb with codeine was explored using the Agilent potentiostat, which measures the voltage, current, and resistance in the electric circuit from the strip. The as-fabricated strips were tested with the potentiostat without immersion in the test solutions, and very low-voltage responses were recorded.

### Using Bromophenol Blue-based Strip

Using the as-fabricated strip coated with various concentrations of Bpb (0.025 mg/mL, 0.05 mg/mL, 0.1 mg/mL, 0.15 mg/mL, and 0.2 mg/mL), the detection of codeine phosphate was performed. The voltage responses were measured with the strip by connecting the copper leads without immersing them in the codeine phosphate solution. It shows that very low voltage responses are due to the absence of analyte solution, and subsequently, each concentration of Bpb-coated strip was immersed in the codeine solution (0.2 mg/mL), and their corresponding voltage responses were tabulated at 0 h (Table S1). To justify the obtained results, the voltage responses of the as-fabricated strips were measured after 24 h, and the results show that there was consistent with the observed voltage responses up to 24 h (Table S2). The potentiometric response is measured at 0 h and 24 h and compared to evaluate the short-term signal stability and the signal drift of the sensing materials. The increase in voltage response with increasing BPB concentration (Table S1) can be attributed to the higher density of charge transfer sites available for interaction with codeine molecules, resulting in enhanced ion exchange and a stronger potentiometric signal. The Bpb concentration was selected from various preliminary optimization studies. Lower concentration showed a poor signal due to less charge transfer, whereas higher concentration resulted in signal saturation. The selected concentration provided a better signal and sensitivity. The calibration plot was constructed using five various concentrations of 0.025 mg/m, 0.05 mg/m, 0.1 mg/m, 0.15 mg/m, and 0.2 mg/mL. Each concentration was measured in triplicate (*n* = 3), and the average potential values were plotted against the logarithm of concentration (log C, mg/mL) to obtain the calibration curve. Standard deviation was represented as error bars. The linear plot of the voltage response versus log concentration was taken at 0 h, and the calculated LoD of Codeine phosphate is 0.0029 mg/mL, with good linearity (R² = 0.992) (Fig. S3a). and after 24 h, the LoD of Codeine phosphate is 0.0022 mg/mL with good linearity (R² = 0.992) (Fig. S3b) (Table S8).

Additionally, the as-fabricated strip coated with Bpb (0.2 mg/mL) was used to detect various concentrations of codeine phosphate (0.025 mg/mL, 0.05 mg/mL, 0.1 mg/mL, 0.15 mg/mL, and 0.2 mg/mL) solution. These concentrations were prepared by the serial dilution method from the cough syrup solution. Each concentration was measured in triplicate (*n* = 3), and the average potential values were plotted against the logarithm of concentration (log C, mg/mL) to obtain the calibration curve. Standard deviation was represented as error bars. Very low voltage responses were observed when the strip was not immersed in the solution. While the strip was immersed in the codeine solutions, the increased voltage responses were measured due to the formation ion-pair complex with the Bpb at 0 h (Table S3). It is noteworthy that the voltage responses were consistently increased with the increase of concentrations of the analyte in the strip within 24 h (Table S4). It confirms the stability of the active material with the interaction of the analyte up to the concentration of 0.0025 mg/mL of codeine phosphate, which was a lower range of detection in clinical settings.

The voltage analysis of the strip prepared with a Single concentration of bromophenol blue (0.2 mg/mL) with various concentrations of codeine phosphate, it is observed that the voltage changes before and after dipping in the codeine Phosphate syrup. As shown in Tables S3 and S4, the voltage increases with an increase in concentration of the codeine phosphate. The linear plot of the voltage response versus log concentration taken at 0 h and 24 h, and the calculated LoD for 0 h (Table S4a) of Codeine phosphate is 0.0037 mg/mL, with good linearity (R² = 0.980), and after 24 h (Table S4b), the LoD of Codeine phosphate is 0.0026 mg/mL with good linearity (R² = 0.994) (Table S8).

### Using Bromophenol Blue Intercalated GO-based Strip

In order to increase the detection of codeine present in the cough syrup solution, it was employed the interaction of Bpb on the surface of GO basal planes was employed through the van der Waals interactions. The active material intercalated GO nanosheets were spin-coated uniformly, and on top of it, a copper electrode was placed. On the other side, the graphite-coated strip with a copper electrode, along with the polyester film as a separator, was developed and sealed together as a strip. The voltage responses show enhanced voltage responses due to the large surface area of the nanosheets. The absence of analyte solution, and subsequently, each concentration of Bpb-coated strip was immersed in the codeine solution (0.2 mg/mL), and their corresponding responses were tabulated at 0 h (Table S5). Each concentration was measured in triplicate (*n* = 3), and the average potential values were plotted against the logarithm of concentration (log C, mg/mL) to obtain the calibration curve. Standard deviation was represented as error bars. For consistency, the experiments were repeated after 24 h (Table S6), and the results are consistent with the previously observed. It confirms the strip prototype detects the codeine phosphate analyte in lower concentrations with reproducibility.

The voltage analysis of the strip prepared with a Single concentration of GO-bromophenol blue (1 mg/mL) with various concentrations of codeine phosphate; it is observed that the voltage changes before and after dipping in the codeine Phosphate syrup. As shown in Tables S5 and S6, the voltage increases with an increase in concentration of the codeine phosphate. The linear plot of the voltage response versus log concentration taken at 0 h and 24 h, and the calculated LoD for 0 h (Fig. S5a) is 0.0018 mg/mL, with good linearity (R² = 0.962), and after 24 h (Fig. S5b), the LoD is 0.0042 mg/mL with good linearity (R² = 0.964) (Table S8).

### Colorimetric analysis of codeine present in the cough Syrup

**Fig. 4:**
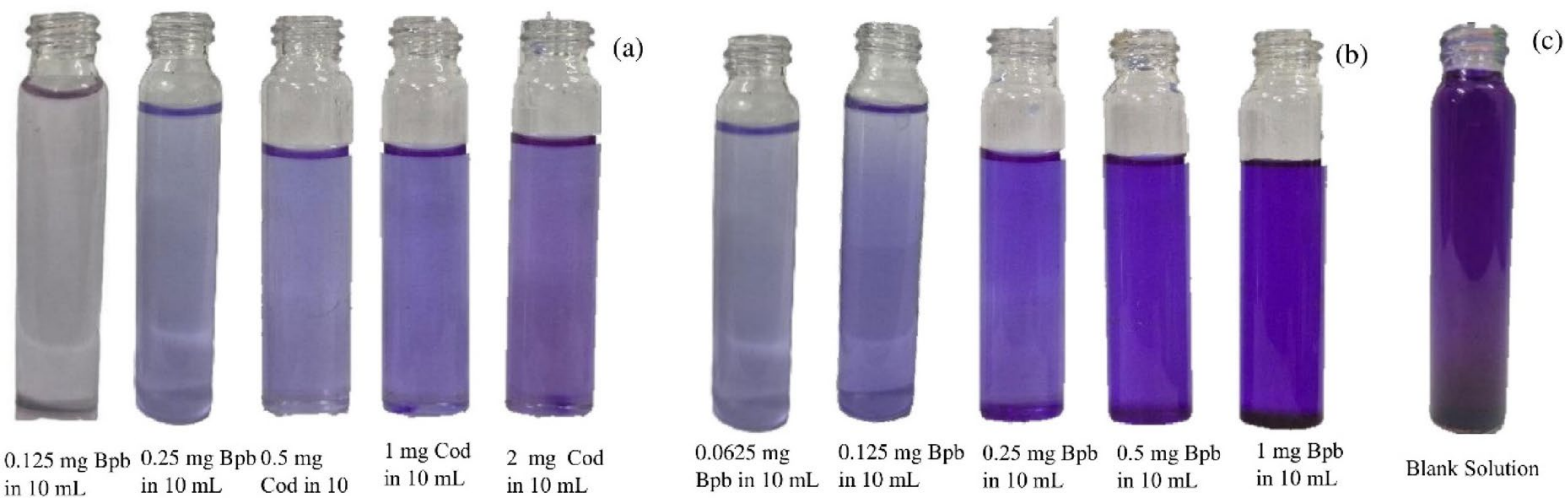
**a** Various concentration of codeine phosphate by taking (0.0125 mg/mL, 0.025 mg/mL, 0.05 mg/mL, 0.1 mg/mL, and 0.2 mg/mL respectively in Distilled Water and mixed with the Single concentration of the 0.025 mg/mL Bpb solution, **b** Various concentration of Bpb (0.00625 mg/mL, 0.0125 mg/mL, 0.025 mg/mL, 0.05 mg/mL, and 0.1 mg/mL ) of deionized water in 0.2 mg/mL of codeine phosphate from cough syrup in deionised water, **c** Blank solution of Bpb.

The detection of codeine phosphate present in the commercial cough syrups was performed by the colorimetric analysis (Fig. 4). The various concentrations of the active molecule bromophenol blue 0.00625 mg/mL, 0.0125 mg/mL, 0.025 mg/mL, 0.05 mg/mL, and 0.1 mg/mL ) were prepared and the sequential colours were developed performed by the addition of codeine phosphate (0.2 mg/mL) to the prepared Bpb solutions. These coloured solutions were analysed by UV-Vis spectroscopy, and their spectra are shown in (Fig. S6a). Five concentration levels (0.00625–0.1 mg/mL) were investigated to evaluate the concentration-dependent absorbance response. The system exhibited linear behavior, and regression was performed within the range of 0.00625–0.1 mg/mL. The resulting calibration curve showed moderate linearity (R² = 0.833). The limit of detection (LoD) was also calculated using the linear calibration plot of absorbance versus concentration (Fig. S6b). Its LoD was calculated as 0.088 mg/mL (Table S8). Similarly, the absorbance of various concentrations of codeine phosphate (0.0125, 0.025, 0.05, 0.1, and 0.2 mg/mL) with the addition of Bpb (0.025 mg/mL) solution was measured (Fig. S6c). The system exhibited linear behavior, and regression was performed within the range of 0.0125–0.2 mg/mL. The resulting calibration curve showed moderate linearity (R² = 0.83). The limit of detection (LoD) was also calculated using the linear calibration plot of absorbance versus concentration (Fig. S6d). Its LoD was calculated as 0.0442 mg/mL (Table S8). The corresponding LoD with various concentrations of codeine phosphate was calculated (Fig. S6d) by the following Eq. (1):

1 $${Limit{\text{ }}of{\text{ }}Detection{\text{ }}\left( {LoD} \right){\text{ }} = \;\;\;\;\frac{{3.3 \times \sigma }}{S}\;}$$

UV Analysis for detecting codeine Phosphate in cough syrup to determine the lower-level detection for the quantitative Analysis. In UV Spectrophotometry, the observation of the result for different concentrations of the codeine phosphate and bromophenol blue (Fig. 4) follows Beer-Lambert’s Law, which states that A = ΕC*l*, A- Absorbance, Ε- Molar Absorption Coefficient, C- Concentration, and *l*-Path Length. The UV spectra of different concentrations of Bpb with codeine show the absorbance in two regions, and the concentration of codeine increases the ion pair interaction, and the corresponding absorbance also increases at the wavelength 200–350 nm, and also the absorbance maxima at 590–600 nm (Fig. S6a).

The efficacy of bromophenol blue intercalated GO nanosheets was explored with the detection and quantification of codeine phosphate cough syrup. Fig. S7a shows the colorimetric response of GO-Bpb to different concentrations of codeine (0.0125 mg/mL, 0.025 mg/mL, 0.05 mg/mL, 0.1 mg/mL, and 0.2 mg/mL) codeine phosphate in the cough syrup. The color intensity was increased steadily as the concentration of the codeine phosphate increased. The system exhibited linear behaviour, and regression was performed within the range of 0.0125–0.2 mg/mL. The resulting calibration curve showed good linearity (R² = 0.956). The limit of detection (LoD) was also calculated using the linear calibration plot of absorbance versus concentration (Fig. S7b), revealing that the quantitative detection of the LoD of codeine phosphate was 0.0412 mg/mL (Table S8).

The accuracy of the strip-based sensor was validated by independently determining the UV-Vis spectrophotometry of codeine phosphate in commercial cough syrup as a reference method. The result measured from the strip using potentiometry was compared with the UV calibration, confirming the reliability of the produced sensing approach for real sample analysis.

Numerous analytical techniques have been developed in the literature for the detection of codeine and its derivatives in pharmaceutical and biological samples, which were summarized and presented in Table 1.

**Table 1:** Reported analytical techniques for the detection of codeine phosphate.

SL. No	Analyte	Detection Technique	Sample Matrix	LoD (mg/mL or ng/mL or nM)	Reference
1	Codeine Phosphate, Diphenhydramine HCl	Zero-Order Derivative UV Spectrophotometry	Cough Mixture	1000 ng/mL	^36^
2	Codeine Phosphate, Paracetamol	First-Order Derivative UV Spectrophotometry	Tablet	260 ng/mL	^37^
3	Codeine	Chromo-amperometry modified screen-printed carbon electrode	Tablet and Urine	4900 nM	^38^
4	Codeine	SWV-Nanodiamond/dihexadecyl Phosphate modified glassy carbon electrode	Tablet, Urine, and Serum	54.5 nM	^39^
5	Codeine	DPV-BDD electrode	Tablet and Urine	80 nM	^40^
6	Codeine Phosphate, Chlorpheniramine maleate	RP-HPLC- UV detector	Oral Syrup	2263 ng/mL	^41^
7	Codeine Phosphate, Paracetamol	RP-HPLC- UV-Vis detector	Tablet	60 ng/mL	^42^
8	Codeine phosphate, Triprolidine Hydrochloride, Pseudoephedrine hydrochloride	RP-HPLC- UV detector	Liquid Formulation	54 ng/mL	^43^
9	Codeine phosphate, Triprolidine Hydrochloride,	Potentiometric and Colorimetric Method	Commercial Cough Syrup	0.0018 mg/mL	Our work

## Conclusion

A simple, low-cost, and portable strip-based sensor was successfully developed for the rapid detection of codeine phosphate, demonstrating a detection limit of 0.0018 mg/mL. The strip-based sensor with the GO-bromophenol blue (Bpb) nanosheets was fabricated with the copper electrode and Teflon separator. The active materials were thoroughly characterized using P-XRD, IR, UV, and SEM techniques to confirm the intercalation and structural integrity of the nanosheets. The integration of graphene oxide intercalated with Bpb significantly enhances the sensor’s sensitivity compared to a non-functionalised Bpb strip and UV-based detection, allowing for trace-level detection, with a limit of detection (LoD) of 0.0018 mg/mL, for detection suitable for potential forensic applications. Designed for point-of-care use, the sensor operates without the need for complex instrumentation or skilled personnel, making it highly suitable for field deployment. This method of detection offers a promising platform for real-time detection of codeine present in the analyte and can be further extended to identify other abused or controlled substances in the process of crime scene investigation. In comparison with previously reported analytical techniques, the strip with GO-Bpb is simple, cost-efficient, and portable. It provides rapid response for on-site forensic detection. It doesn’t require any sophisticated instrumentation and is easily accessible for preliminary studies. However, the proposed approach has limitations, such as sensitivity is lower sensitivity than other chromatographic techniques, a lack of selectivity towards structurally similar compounds, and long-term stability and sensing for a wide range of real samples.

## Supplementary Information

Below is the link to the electronic supplementary material.

Supplementary Material 1

## Data Availability

All data generated or analyzed during this study are included in this published article and its supplementary information files.
