# Supplementary material for: Functionalized graphene oxide-based electrode material for the potentiometric detection of codeine phosphate in commercial cough syrups for forensic applications
Source: Sci Rep. 2026 Apr 8;16:16666. doi: 10.1038/s41598-026-44986-4 (PMC13219407; doi:10.1038/s41598-026-44986-4)
Supplement: Supplementary file 1 — Supplementary Material 1 [file 41598_2026_44986_MOESM1_ESM.docx]

**Functionalized Graphene Oxide Based Electrode Material for the Potentiometric Detection of Codeine Phosphate Present in the Commercially Available Cough Syrups for Forensic Applications**

Ephrin S^1^, Jebasingh Bhagavathsingh^1,2*^, Sneha Abraham^3^, Nesasudha M^4^, Doondi Kumar Janapala^5*^

^1^Department of Applied Chemistry, Karunya Institute of Technology and Sciences, Coimbatore-641114, Tamilnadu, INDIA

^2^Department of Chemistry, School of Energy Technology, Pandit Deendayal Energy University, Raysan, Gandhinagar, Gujarat-382426, INDIA

^3^Centre for Nanosciences and Genomics, Karunya Institute of Technology and Sciences, Coimbatore-641114, Tamilnadu, INDIA

^4^Department of Electronics and Communication Engineering, Karunya Institute of Technology and Sciences, Coimbatore-641114, Tamilnadu, INDIA

^5^Department of Electronics and Communication Engineering, Vishnu Institute of Technology, Bhimavaram-534202, Andra Pradesh, INDIA

Corresponding Author’s Email: [B.Jebasingh@spt.pdpu.ac.in,](mailto:B.Jebasingh@spt.pdpu.ac.in) and [doondi.j@vishnu.edu.in](mailto:doondi.j@vishnu.edu.in)

**Supplementary Information**

**TABLE OF CONTENTS**

| **S. No** | **Title** | **Page No.** |
| --- | --- | --- |
| **1.** | **Fig. S1** SEM images of (a) pristine GO (scale bar: 20 µm), and GO–Bpb at different scales: (b) high-resolution image (scale bar: 200 nm) (c) intermediate-scale image (scale bar: 2 µm) and (d) low-magnification image (scale bar: 20 µm) | **S2** |
| **2.** | **Fig. S2** SEM–EDX spectrum of GO–Bpb | **S2** |
| **3.** | **Fig. S3** Calibration curves of potentiometric response (mV) versus Bpb concentrations (log C, mg/mL) at 0 h and 24 h. Error bars indicate standard deviation (n = 3) | **S3** |
| **4.** | **Fig. S4** Calibration curves of potentiometric response (mV) versus codeine phosphate (log C, mg/mL) at 0 h and 24 h. Error bars indicate standard deviation (n = 3) | **S3** |
| **5.** | **Fig. S5** Calibration curves of potentiometric response (mV) versus codeine phosphate (log C, mg/mL) with GO-Bpb coated Strip at 0 h and 24 h. Error bars indicate standard deviation (n = 3) | **S3** |
| **6.** | **Fig. S6** **a** UV-Visible spectra of the various concentration of Bpb (0.00625 mg/mL, 0.0125 mg/mL, 0.025 mg/mL, 0.05 mg/mL, and 0.1 mg/mL) and the codeine phosphate concentration of 0.2 mg/mL, **b** linear calibration plot of codeine phosphate detection in various concentration of Bpb, **c**  various concentration of codeine phosphate (0.0125 mg/mL, 0.25 mg/mL, 0.05 mg/mL, 0.1 mg/mL, and 0.2 mg/mL) with the BPB (0.025 mg/mL) and **d** Linear calibration plot for detection of various concentration of codeine phosphate of Bpb (0.2 mg/mL) | **S4** |
| **7.** | **Fig. S7 a** UV-Visible spectra of codeine phosphate concentrations (0.0125 mg/mL, 0.025 mg/mL, 0.05 mg/mL, 0.1 mg/mL, and 0.2 mg/mL) and BPB intercalated GO nanosheets (0.2 mg/mL), **b** Calibration plot of various concentrations of codeine phosphate and the concentration of bromophenol blue intercalated GO nanosheets | **S4** |
| **8.** | **Table S1** Potentiometric response of Codeine Phosphate (0.2 mg/mL) in the cough syrup using various concentrations of Bpb-coated strip at 0 h (n=3). | **S5** |
| **9.** | **Table S2** Potentiometric response of Codeine Phosphate (0.2 mg/mL) in the cough syrup using various concentrations of Bpb-coated strip at 24 h (n=3). | **S5** |
| **10.** | **Table S3** Potentiometric response to various concentrations of codeine phosphate using a Bpb-coated strip (0.2 mg/mL) at 0 h (n=3). | **S5** |
| **11.** | **Table S4** Potentiometric response to various concentrations of codeine phosphate using a Bpb-coated strip (0.2 mg/mL) at 24 h (n=3). | **S6** |
| **12.** | **Table S5** Potentiometric response of codeine phosphate in cough syrup using bpb Intercalated GO (1mg/mL) coated strip at 0 h (n=3). | **S6** |
| **13.** | **Table S6** Potentiometric response of codeine phosphate in cough syrup using BPB Intercalated GO (1mg/mL) coated strip at 24 h (n=3). | **S6** |
| **14.** | **Table S7** Elemental composition of GO-Bpb. | **S7** |
| **15.** | **Table S8** Limit of Detection (LoD) Calculation of codeine phosphate | **S7** |

**Fig. S1** SEM images: a pristine GO (scale bar: 20 µm), and GO–Bpb at different scales, **b** high-resolution image (scale bar: 200 nm), **c** intermediate-scale image (scale bar: 2 µm) and **d** low-magnification image (scale bar: 20 µm)


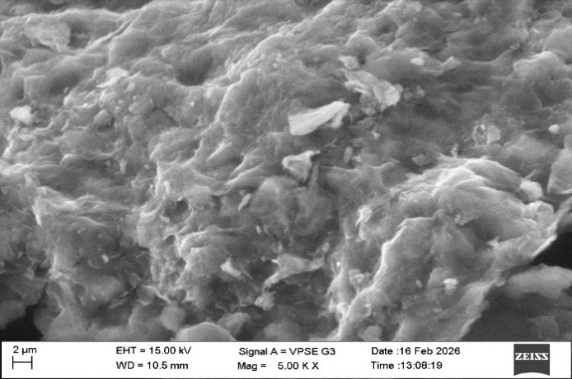

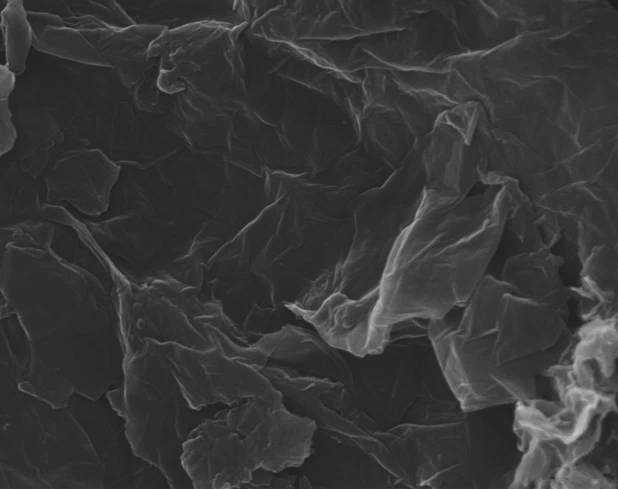


**20 µm**


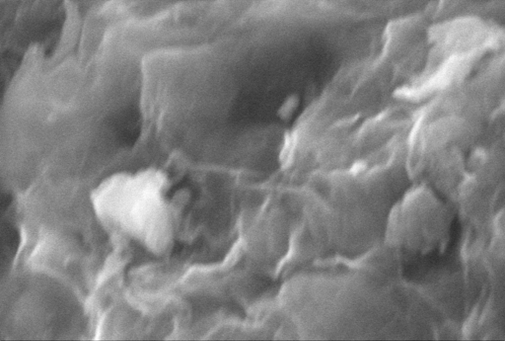


**200 nm**

**(a)**

**(b)**


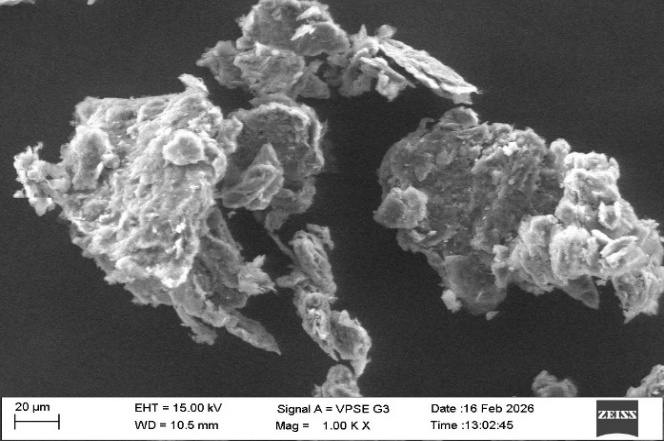


**(c)**

**(d)**

**20 µm**

**2 µm**


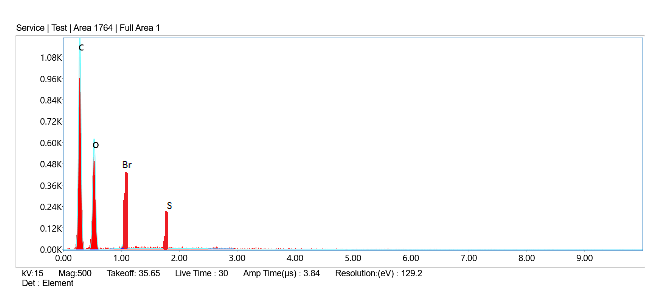
**Fig. S2** SEM–EDX spectrum: **a** SEM of the analysed region and **b** EDX spectrum

(a)

(b)

**100 µm**


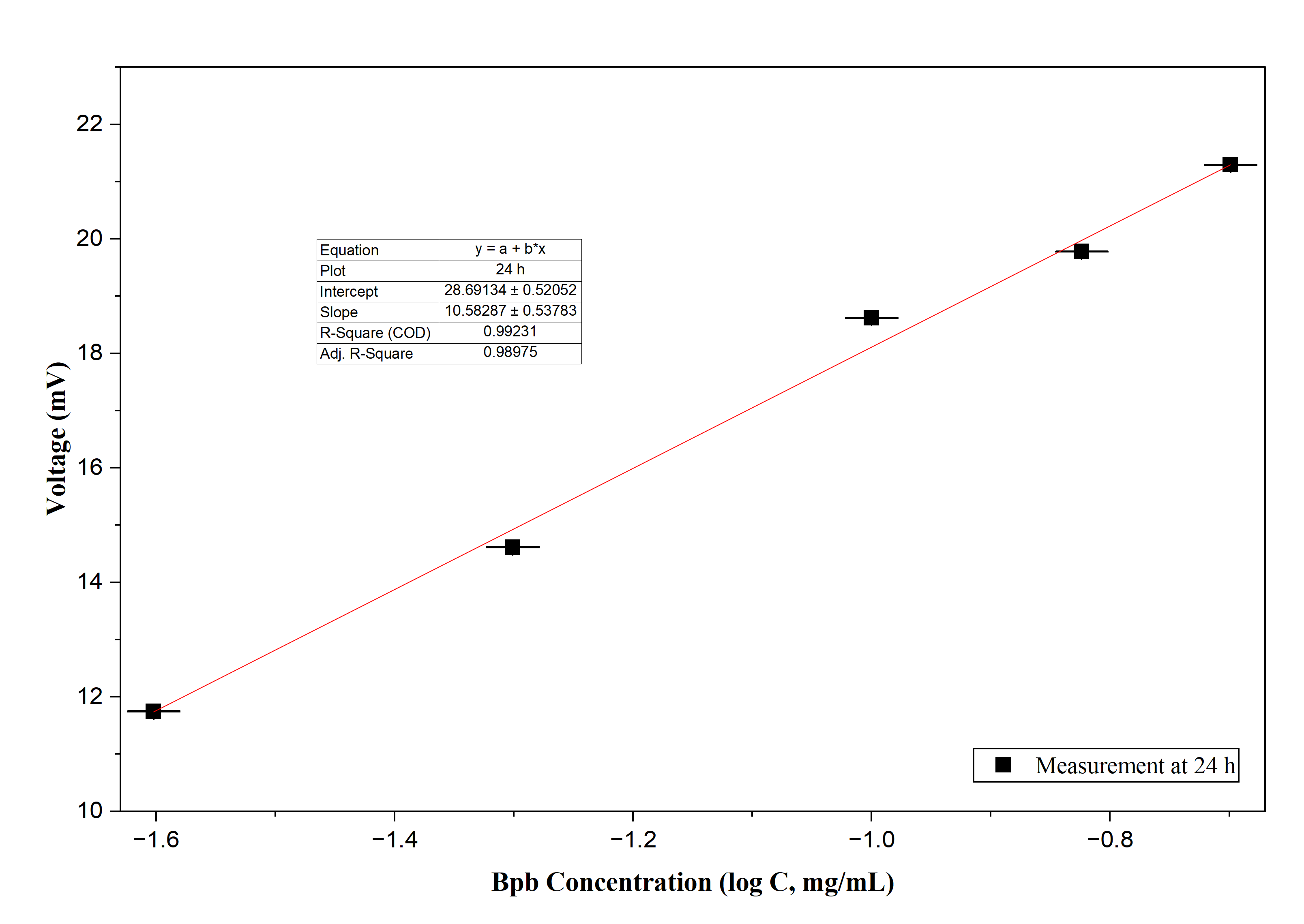

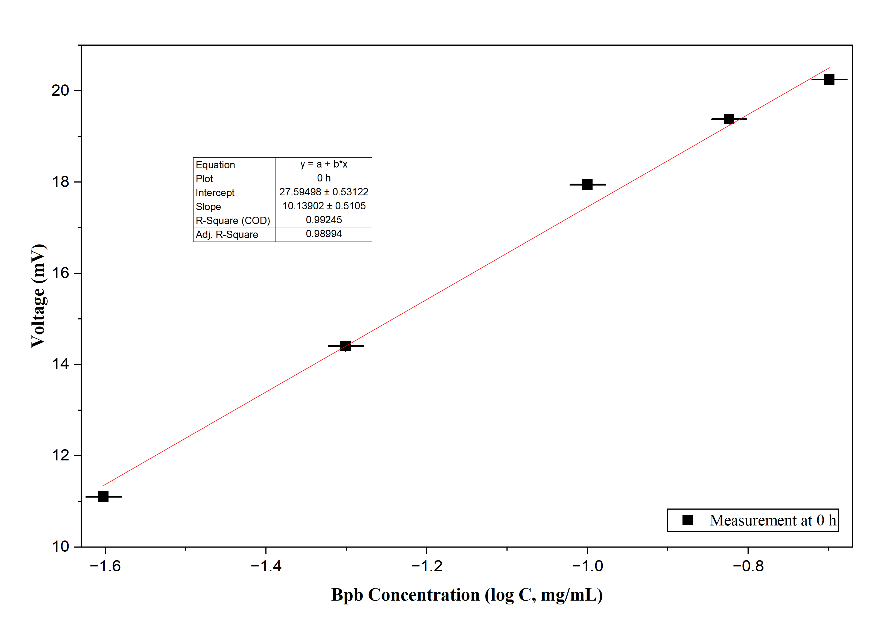
**Fig. S3** Calibration curves of potentiometric response (mV) versus Bpb concentrations (log C, mg/mL) at 0 h and 24 h. Error bars indicate standard deviation (n = 3)

(b)

(a)

**
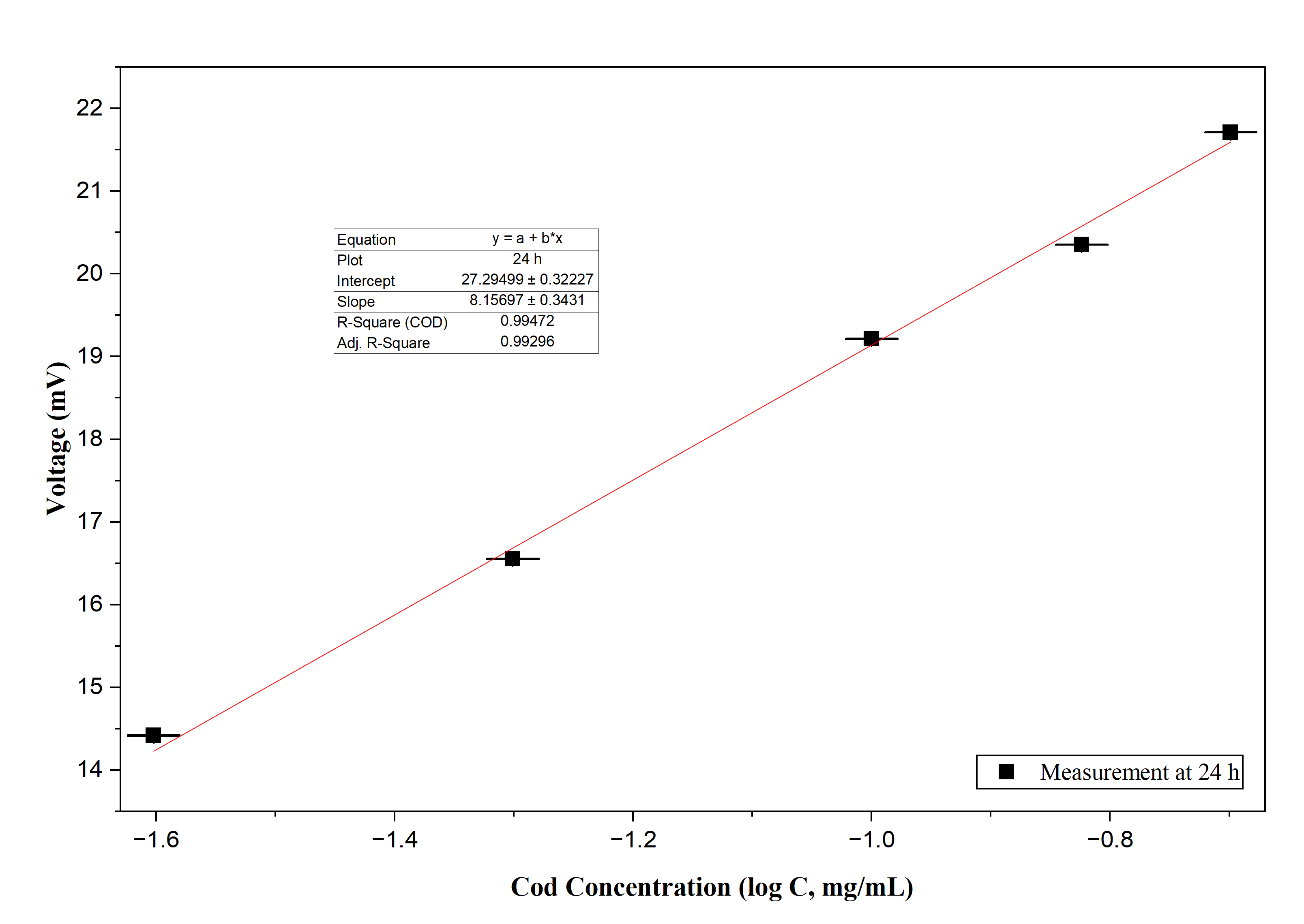
**
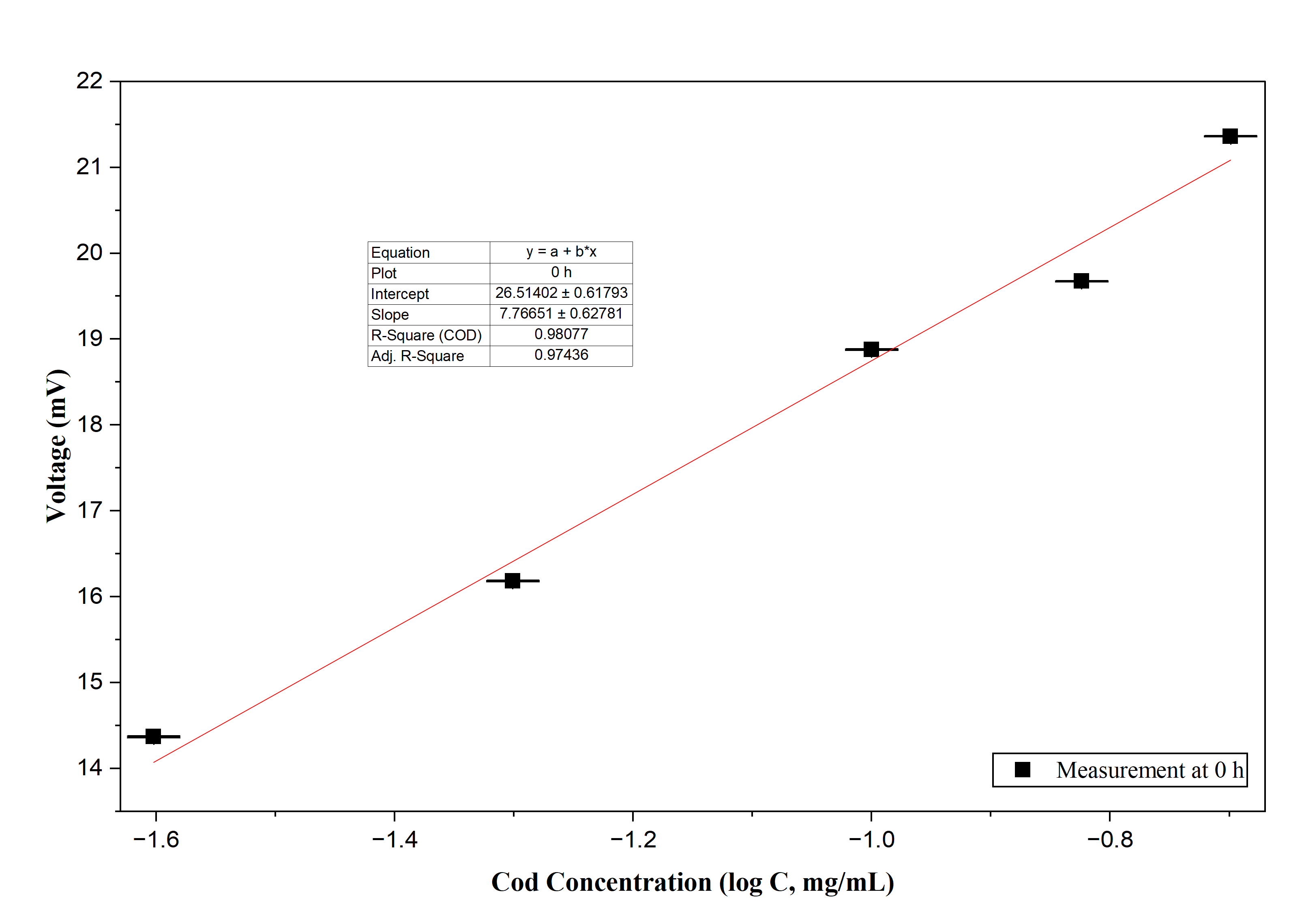
**Fig. S4** Calibration curves of potentiometric response (mV) versus codeine phosphate (log C, mg/mL) at 0 h and 24 h. Error bars indicate standard deviation (n = 3)

(a)

(b)


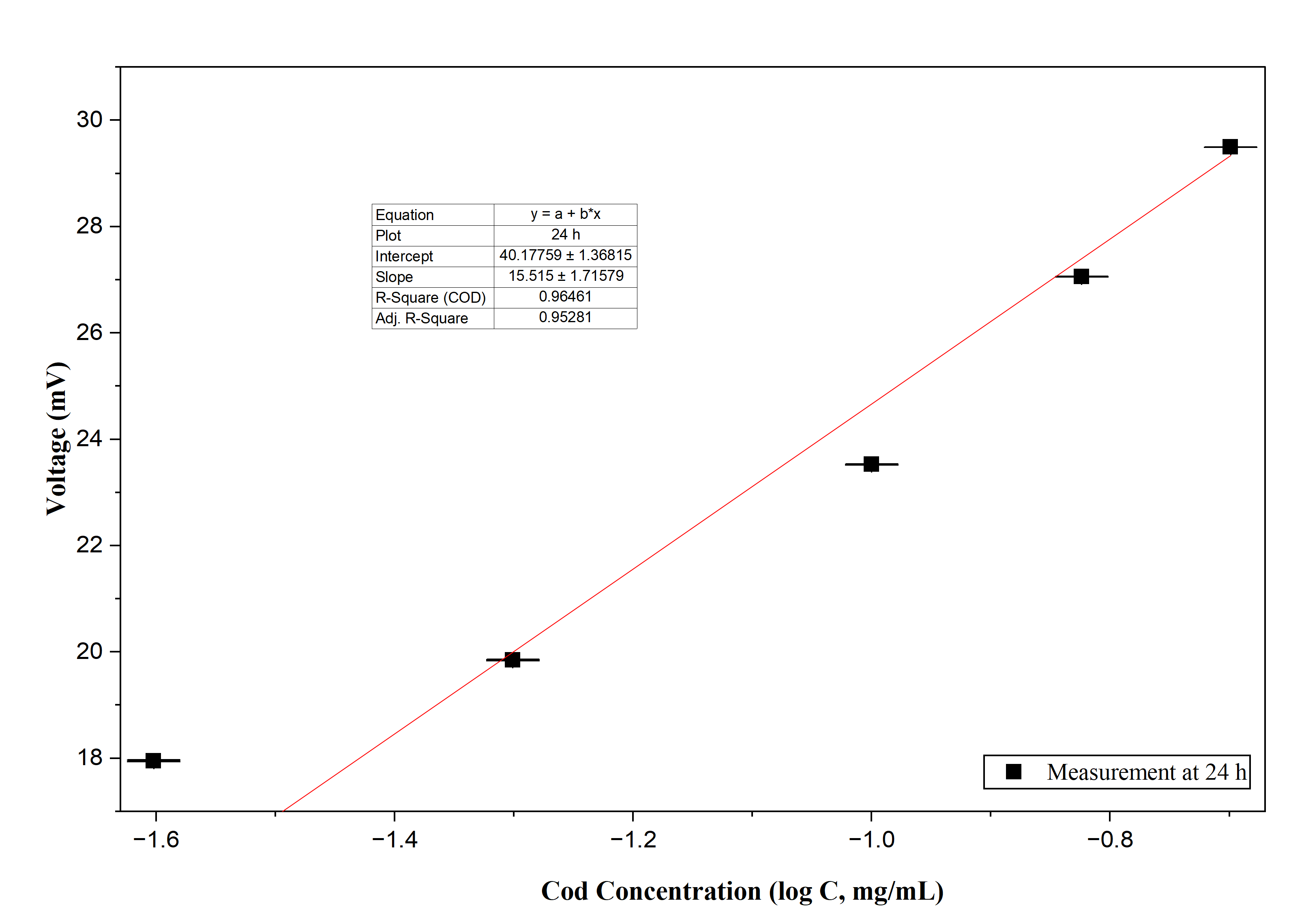
**
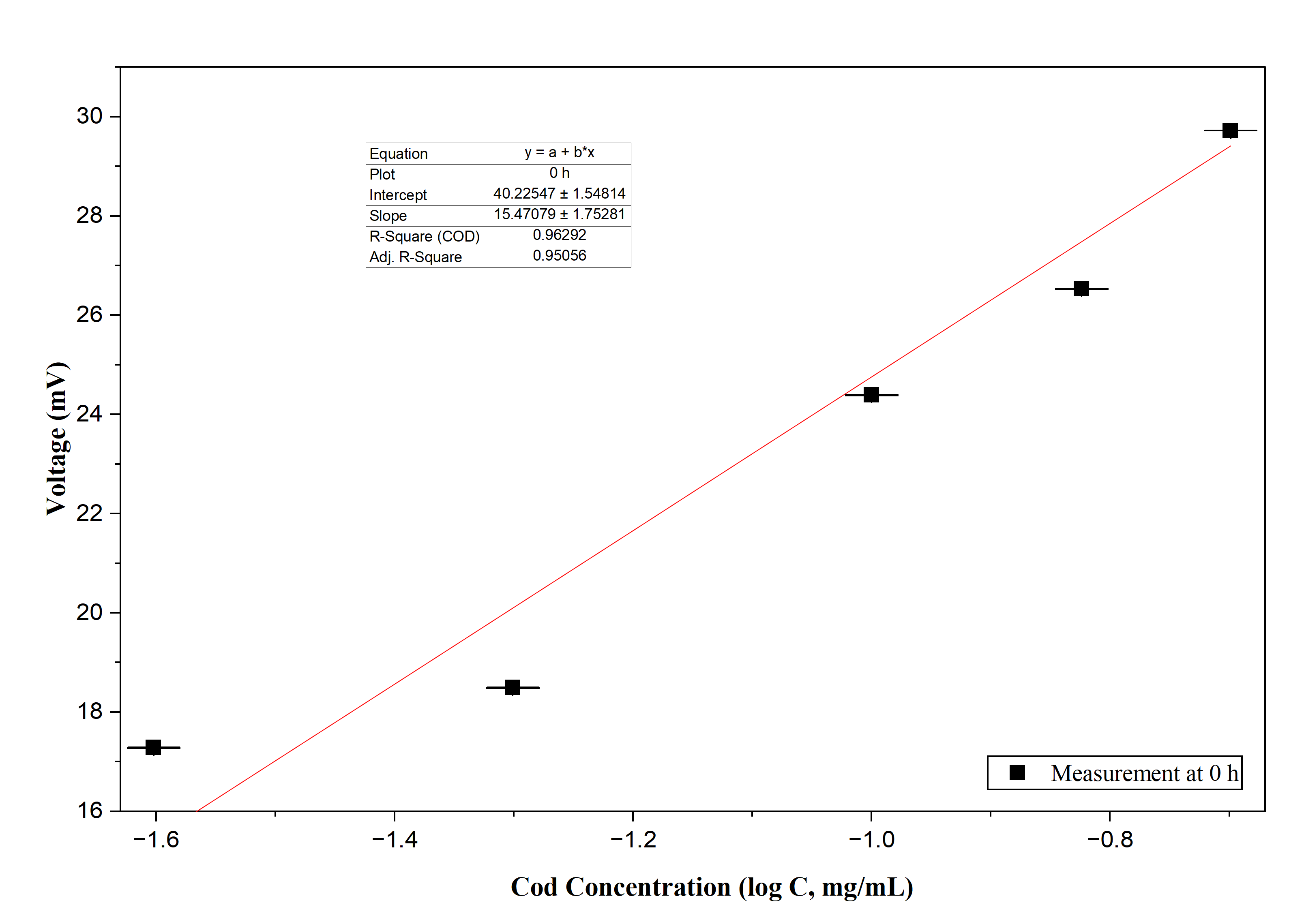
Fig. S5** Calibration curves of potentiometric response (mV) versus codeine phosphate (log C, mg/mL) with GO-Bpb coated Strip at 0 h and 24 h. Error bars indicate standard deviation (n = 3)

(b)

(a)

**Fig. S6** **a** UV-Visible spectra of the various concentration of Bpb (0.00625 mg/mL, 0.0125 mg/mL, 0.025 mg/mL, 0.05 mg/mL, and 0.1 mg/mL) and the codeine phosphate concentration of 0.2 mg/mL , **b** linear calibration plot of codeine phosphate detection in various concentration of Bpb, **c**  various concentration of codeine phosphate (0.0125 mg/mL, 0.25 mg/mL, 0.05 mg/mL, 0.1 mg/mL, and 0.2 mg/mL) with the Bpb(0.025 mg/mL) and **d** Linear calibration plot for detection of various concentration of codeine phosphate of Bpb (0.2 mg/mL)


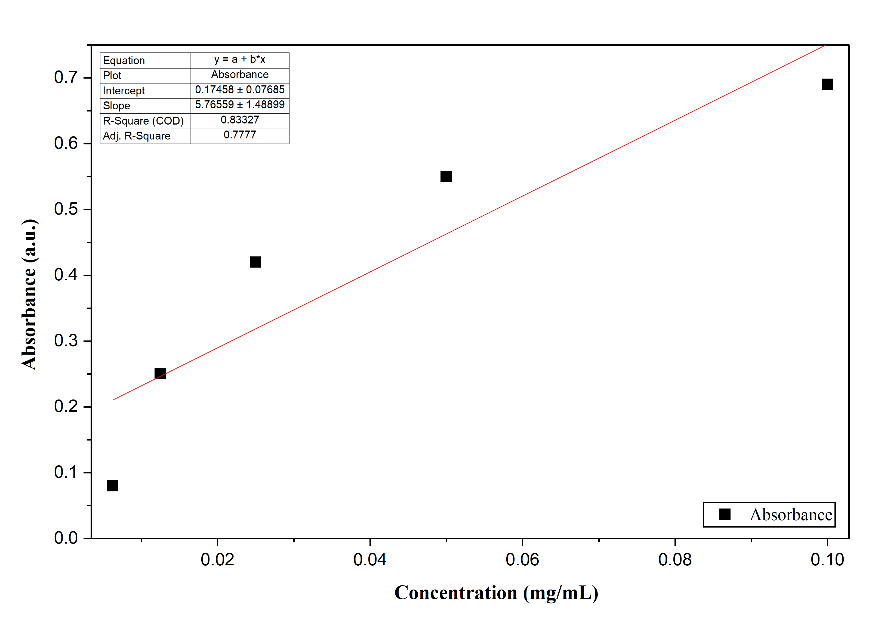

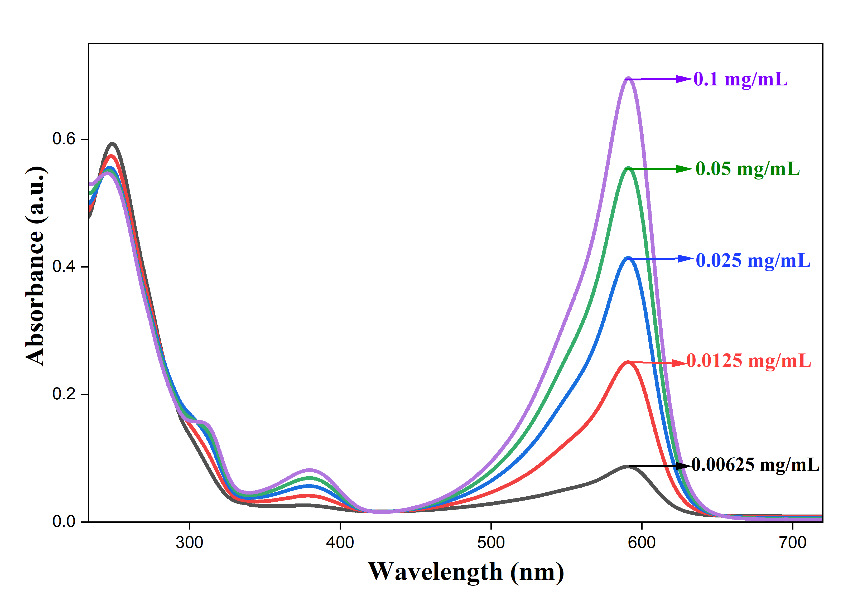


(a)

(b)

**
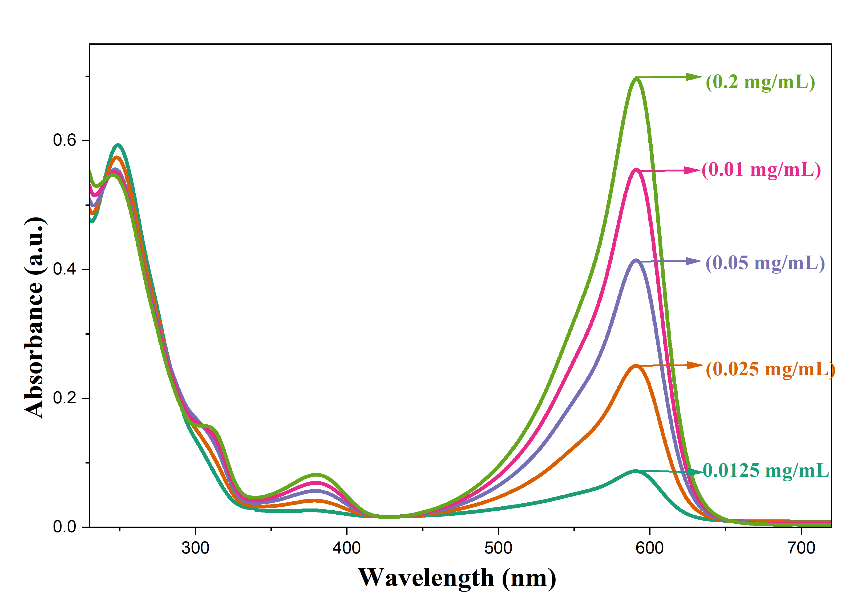
**


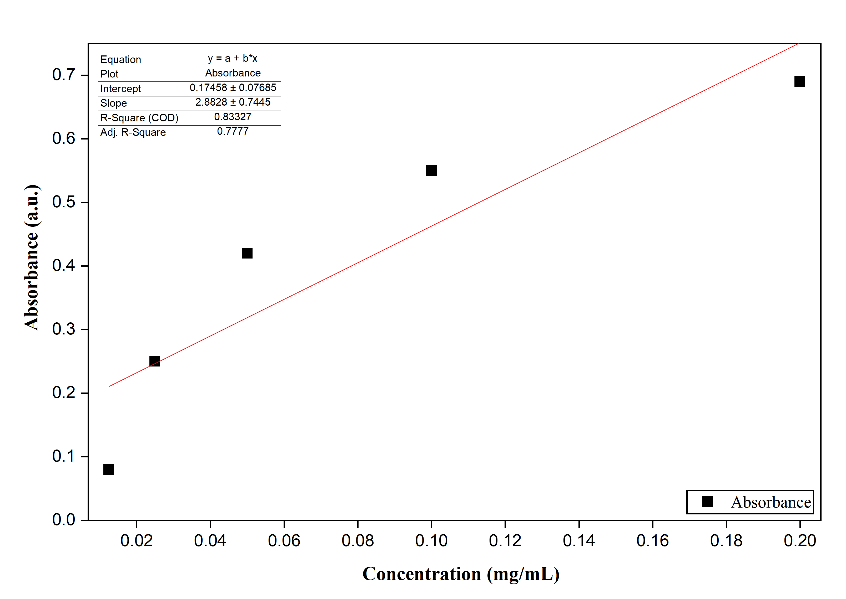


(c)

(d)


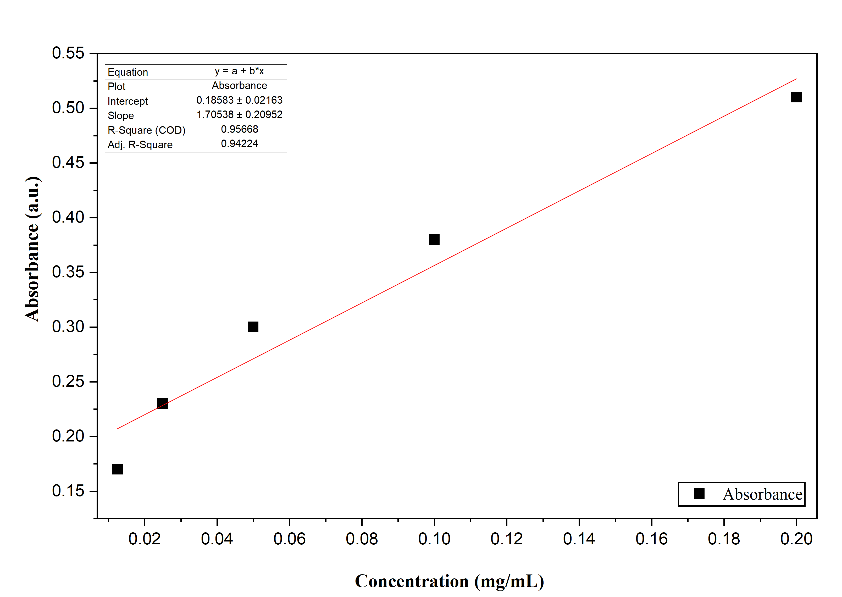
**
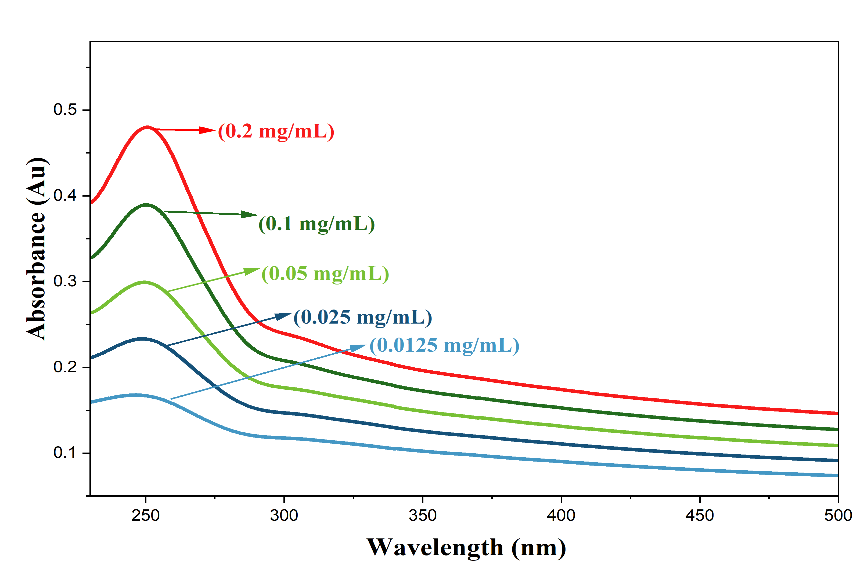
Fig. S7** **a** UV-Visible spectra of codeine phosphate concentrations (0.0125 mg/mL, 0.025 mg/mL, 0.05 mg/mL, 0.1 mg/mL, and 0.2 mg/mL) and Bpb intercalated GO nanosheets (0.2 mg/mL), **b** Calibration plot of various concentrations of codeine phosphate and the concentration of bromophenol blue intercalated GO nanosheets

(b)

(a)

**Table S1**. Potentiometric response of Codeine Phosphate (0.2 mg/mL) in the cough syrup using various concentrations of Bpb-coated strip at 0 h (n=3).

*Measurements performed in triplicate (n = 3).

**Table S2** Potentiometric response of Codeine Phosphate (0.2 mg/mL) in the cough syrup using various concentrations of Bpb-coated strip at 24 h (n=3).

*Measurements performed in triplicate (n = 3).

**Table S3** Potentiometric response of various concentrations of codeine phosphate using Bpb-coated strip (0.2 mg/mL) at 0 h (n=3).

*Measurements performed in triplicate (n = 3).

**Table S4**. Potentiometric response to various concentrations of codeine phosphate using a Bpb-coated strip (0.2 mg/mL) at 24 h (n=3).

*Measurements performed in triplicate (n = 3).

**Table S5.** Potentiometric response of codeine phosphate in cough syrup using Bpb Intercalated GO (1mg/mL) coated strip at 0 h (n=3).

*Measurements performed in triplicate (n = 3).

**Table S6** Potentiometric response of codeine phosphate in cough syrup using Bpb Intercalated GO (1mg/mL) coated strip at 24 h (n=3).

*Measurements performed in triplicate (n = 3).

| Element | Weight % | Atomic % |
| --- | --- | --- |
| C K | **32.3** | **42.6** |
| O K | **45.1** | **41.8** |
| Br K | **14.7** | **10.1** |
| S K | **7.9** | **5.5** |

**Table S7** Elemental composition of GO-Bpb.

| **Table S8** Limit of Detection (LoD) Calculation of codeine phosphate   \| Sl. No \| Method \| LOD Calculation \| \| --- \| --- \| --- \| \| 1. \| UV-Visible spectra of Various concentrations of Bpb (0.00625 mg/mL, 0.0125 mg/mL, 0.025 mg/mL, 0.05 mg/mL, and 0.1 mg/mL) and the codeine phosphate concentration of 0.2 mg/mL. \| LoD of Cod-0.0881 mg/mL \| \| 2. \| UV-Visible spectra of Codeine Phosphate concentration (0.0125 mg/mL, 0.025 mg/mL, 0.05 mg/mL, 0.1 mg/mL, and 0.2 mg/mL) and Bpb concentration (0.025 mg/mL) \| LoD of Cod-0.0442 mg/mL \| \| 3. \| UV-Visible spectra of codeine phosphate concentrations (0.0125 mg/mL, 0.025 mg/mL, 0.05 mg/mL, 0.1 mg/mL, and 0.2 mg/mL) and Bpb intercalated GO nanosheets (0.2 mg/mL) \| LoD of Cod-0.0412 mg/mL \| \| 4. \| Voltage Analysis of Codeine Phosphate in Cough Syrup using various concentrations of Bpb based Strip \| LoD of Cod (at 0 h)-0.0029 mg/mL  LoD of Cod (at 24 h)-0.0022 mg/mL \| \| 5. \| Voltage Analysis of various concentrations of Codeine Phosphate in Cough Syrup using Bpb Strip (0.2 mg/mL) \| LoD of Cod (at 0 h)-0.0037 mg/mL  LoD of Cod (at 24 h)- 0.0026 mg/mL \| \| 6. \| Voltage Analysis of Codeine Phosphate in Cough Syrup using Bpb Intercalated GO (1 mg/mL) based Strip \| LoD of Cod (at 0 h)-0.0018 mg/mL  LoD of Cod (at 24 h)-0.0042 mg/mL \| |
| --- | --- | --- | --- | --- | --- | --- | --- | --- | --- | --- | --- | --- | --- | --- | --- | --- | --- | --- | --- | --- | --- |
